# Enhanced hybrid photocatalytic dry reforming using a phosphated Ni-CeO_2_ nanorod heterostructure

**DOI:** 10.1038/s41467-023-36982-3

**Published:** 2023-03-15

**Authors:** Alexandra Tavasoli, Abdelaziz Gouda, Till Zähringer, Young Feng Li, Humayra Quaid, Camilo J. Viasus Perez, Rui Song, Mohini Sain, Geoffrey Ozin

**Affiliations:** 1 Department of Chemistry, University of Toronto, 80 St. George Street, M5S 3H6 Toronto, Canada; 2 Department of Materials Science & Engineering, University of Toronto, 184 College St., M5S 3E4 Toronto, Canada; 3 Department of Mechanical and Industrial Engineering, University of Toronto, 5 King’s College Rd, Toronto, ON M5S 3G8 Canada

**Keywords:** Photocatalysis, Heterogeneous catalysis

## Abstract

Operating the dry reforming reaction photocatalytically presents an opportunity to produce commodity chemicals from two greenhouse gases, carbon dioxide and methane, however, the top-performing photocatalysts presented in the academic literature invariably rely on the use of precious metals. In this work, we demonstrate enhanced photocatalytic dry reforming performance through surface basicity modulation of a Ni-CeO_2_ photocatalyst by selectively phosphating the surface of the CeO_2_ nanorod support. An optimum phosphate content is observed, which leads to little photoactivity loss and carbon deposition over a 50-hour reaction period. The enhanced activity is attributed to the Lewis basic properties of the PO_4_^3−^ groups which improve CO_2_ adsorption and facilitate the formation of small nickel metal clusters on the support surface, as well as the mechanical stability of CePO_4_. A hybrid photochemical-photothermal reaction mechanism is demonstrated by analyzing the wavelength-dependent photocatalytic activities. The activities, turnover numbers, quantum efficiencies, and energy efficiencies are shown to be on par with other dry-reforming photocatalysts that use noble metals, representing a step forward in understanding how to stabilize ignoble nickel-based dry reforming photocatalysts. The challenges associated with comparing the performance of photocatalysts reported in the academic literature are also commented on.

## Abstract (web summary)

The top-performing dry reforming photocatalysts in the literature rely on the use of precious metals. Here, enhanced photocatalytic dry reforming performance is reported through surface basicity modulation of a Ni/CeO_2_ photocatalyst, achieved by selectively phosphating the surface of a CeO_2_ nanorod support.

## Introduction

Operating the dry reforming reaction (CO_2_ + CH_4_ ⇌ 2H_2_ + 2CO) photocatalytically can solve several challenges facing the sustainability transition of the commodity chemicals sector. First, dry reforming of methane (DRM) can enable certain green chemical production pathways in the circular chemical economy as carbon dioxide (CO_2_) and (CH_4_) can be sourced from the anaerobic digestion of organic wastes, or captured from emission points to produce synthesis gas, the mixture of hydrogen (H_2_) and carbon monoxide (CO) gas that results from DRM^1^ and is used industrially to produce megaton-scale commodity chemicals^2^ Second, making use of sunlight to drive DRM photocatalytically can replace or reduce the amount of thermal or electrical energy required to drive the reaction, which itself has high energy demands due to both high endothermicity (ΔH_rxn_ = 247.3 kJ mol^−1^) and endergonic nature below temperatures of approximately 700 °C. When these energy requirements are met in the traditional manner by supplying heat from fossil fuel combustion, the potential carbon savings afforded by consuming CO_2_ as feedstock are negated^3^ Electrical heating using renewable electricity is a possibility to alleviate this issue; however, it has been shown that the US electrical grid would need to be not only retrofitted to be emission-free, but the capacity would have to be increased by almost 75% (from 15 to 26 EJ in the United States) to meet the current thermal energy demands of the industrial sector^4^ Because of these potential benefits, using sunlight to assist the dry reforming reaction via photocatalytic pathways is an attractive proposition to aid in the energy transition and has received significant attention in the last two decades.

In addition to the high energetic demands of the dry reforming reaction, it faces operational challenges due to its complex reaction network, which can limit the selectivity of the system towards the desired dry reforming reaction. This deviation from ideal selectivity is observable in the H_2_:CO ratio in the product gas mixture, as well as through the propensity of the dry reforming reaction system to produce solid carbon that leads to catalyst deactivation. The reverse water gas shift reaction (CO_2_ + H_2_ ⇌ CO + 2H_2_O) is thermodynamically favored over the main dry reforming reaction at temperatures below ~700 °C, and leads to the consumption of H_2_ and increased production of CO and can lead to H_2_:CO ratios in the product gas mixture of less than 1 (See Supplementary Information: Fig. S1 and Table S1). Similarly, another potential competing reaction is the steam methane reforming reaction (CH_4_ + H_2_O ⇌ CO + 3H_2_) which has the potential to consume water produced and increase the H_2_:CO ratio in the product gas mixture. Solid carbon can be deposited onto the surface of the photocatalyst by two main avenues: pyrolysis (CH_4_ ⇌ C + 2H_2_) and the Boudouard reaction (2CO ⇌ CO_2_ + C), which both consume carbon monoxide and can increase the H_2_:CO ratio in the product gas mixture. The Boudouard reaction is thermodynamically favorable at temperatures below approximately 700 °C, while pyrolysis is the thermodynamically favored pathway at higher temperatures.

To date, a substantial portion of the work on the photocatalytic dry reforming reaction has focused on the development of more advantageous catalyst materials that can serve to alleviate these economic drawbacks. Photocatalyst compositions, including a CeO_2_ component, have been particularly dominant in the academic literature surrounding the development of dry-reforming photocatalysts, due to the well-documented ability of CeO_2_ to mitigate photocatalyst deactivation by virtue of the Ce^3+^/Ce^4+^ redox couple which enables the material to shuttle oxygen through its lattice and oxidize adsorbed carbon species to prevent their accumulation^5,6^. Several CeO_2_-based thermal dry reforming catalysts have been reported with long-term stability. A table indexing these literature examples and others, along with their performance metrics, is provided in the Supplementary Information (Table S2).

In the present work, we demonstrate enhanced photocatalytic dry reforming performance through surface basicity modulation of a Ni-CeO_2_ photocatalyst by selectively phosphating the surface of a CeO_2_ nanorod support. An optimum phosphate content is observed, which leads to negligible activity loss and carbon deposition over a 50-h reaction period. The enhanced activity is attributed to the Lewis basic properties of the PO_4_^3−^ groups which improve CO_2_ adsorption and facilitate the formation of small metal clusters on the support surface, while providing mechanical stability. A hybrid photochemical-photothermal reaction mechanism is demonstrated by analyzing the wavelength-dependent photocatalytic activities. The photocatalyst activities, turnover numbers, quantum efficiencies, and energy efficiencies are shown to be on par with other dry-reforming photocatalysts that use noble metals such as Pt, Ru, or Rh, representing a significant step forward for more economical ignoble nickel-based dry reforming photocatalysts. The challenges associated with comparing photocatalysts reported in the academic literature are also commented on.

## Results

### Physical and optical properties of the Ni-CeO_2_-CePO_4_ photocatalyst

**Fig. 1:**
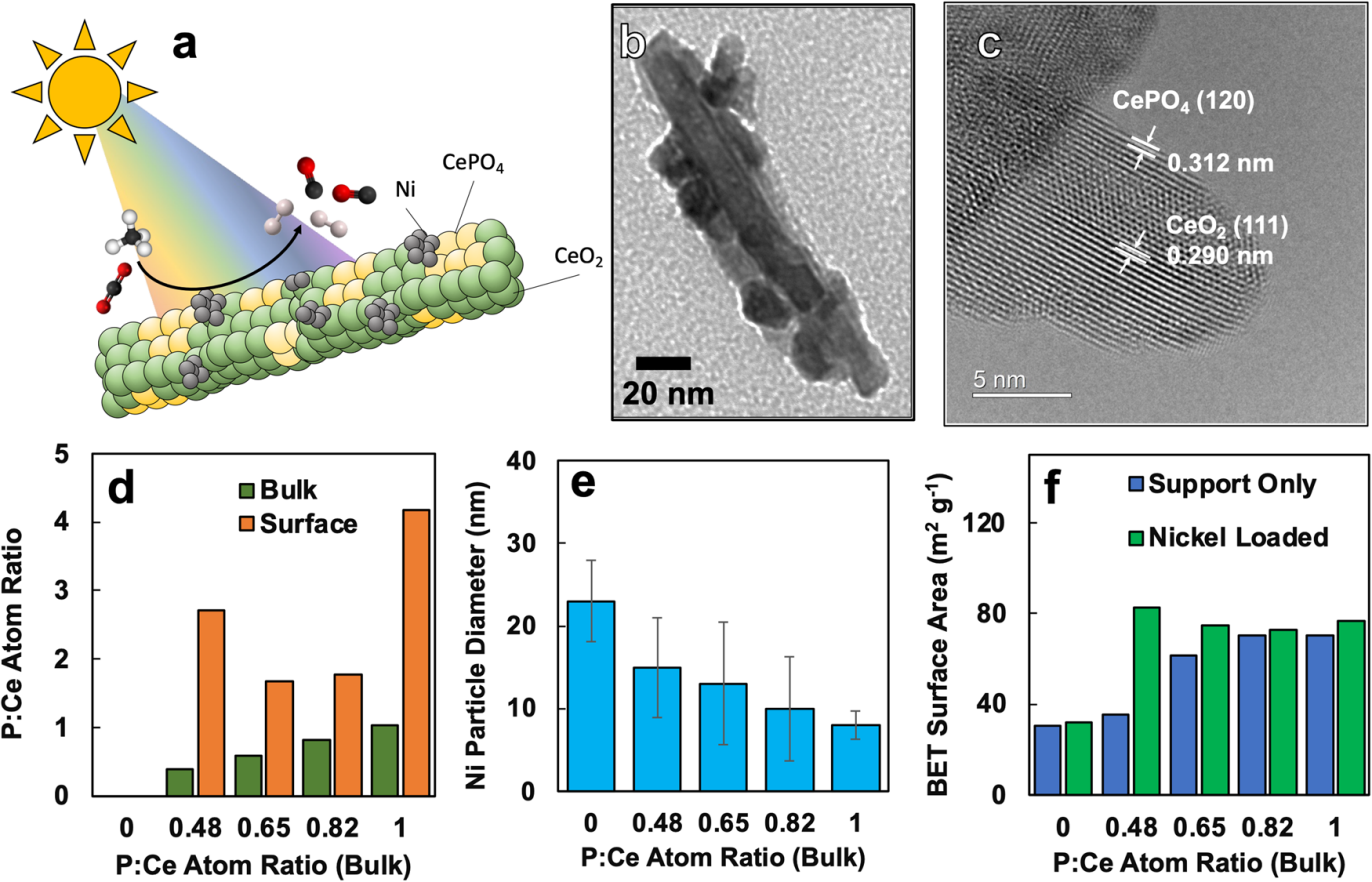
Composition, morphology, and surface area of the Ni-CeO_2_-CePO_4_ photocatalyst featured in the present study. **a** A graphical schematic depicting the structure of the photocatalyst and the dry reforming reaction activated by sunlight; **b** Transmission electron micrograph of the Ni-CeO_2_-CePO_4_, showing the CeO_2_-CePO_4_ nanorod backbone heterostructure decorated with Ni nanoparticles; **c** High-resolution transmission electron micrograph showing the spatial partitioning of CeO_2_ and CePO_4_; **d** Surface partitioning of phosphorous as measured by EDS and XPS; **e** Average diameter of nickel nanoparticles calculated from TEM images, which is shown to decrease with increasing phosphorous addition; and **f** BET surface areas of photocatalyst samples.

Ni-CeO_2_-CePO_4_ photocatalyst samples, whose physical morphology consisted of a nanorod heterostructure comprised of a CeO_2_-CePO_4_ backbone decorated with Ni nanoparticles (Fig. 1b and S2, S3 in the Supplementary Information), were prepared with nominal Ni loadings of 10 at.%. Measurement using energy dispersive x-ray spectroscopy (EDS) revealed actual Ni loadings to be between 1.91 and 2.38 at.%, similar to the other studies using Ni-based dry reforming photocatalysts^7^.

The purpose of this study was to investigate the enhancement in photocatalyst activity and stability that results from the addition of Lewis basic PO_4_^3−^ groups on the photocatalyst support surface. To this end, a range of Ni-CeO_2_-CePO_4_ photocatalyst samples with nominal P:Ce ratios of 0.00, 0.25, 0.50, 0.75, and 1.00 were prepared using a room temperature aqueous synthesis that is described in detail in the Supplementary Information. EDS measured the compositions of the Ni-CeO_2_-CePO_4_ photocatalyst samples to have P:Ce ratios of 0.00, 0.48, 0.65, 0.82, and 1.00, which varied from the nominal ratios listed above but still provided a good distribution over the targeted composition range (Supplementary Information: Table S3). The measured compositions of the catalyst will be used in the discussion below.

X-ray photoelectron spectroscopy (XPS) was performed to characterize and quantify the chemical states present at the surface of the photocatalyst. The Ce 3*d* spectrum shows five components at 881.5 eV (*V*), 885.5 eV (*V’’*), 900.4 eV (*U*), 904.1 eV (*U’’*), and 916.8 eV (*U’’’*) corresponding to the main spectral lines and satellites of Ce^3+^ state of cerium phosphate^8^ The O 1*s* spectra show two peaks at binding energies 530.3 and 532.0 eV.  The dominant peak at lower binding energy (530.3 eV) can be ascribed to lattice oxygen. The higher binding energy peak observed at 532.1 eV may arise from the chemisorbed oxygen or hydroxyl species with increasing phosphorous content. The P 2*p* spectra exhibit one characteristic spectral line located at 133.5 eV (P 2*p*^1/2^), characteristic of pentavalent phosphorus (PO_4_^3−^ group) in the lattice. XPS spectra are available in the accompanying Supplementary Information: Fig. S4 and Table S3.

Comparative inspection of the surface and bulk atom ratios of phosphorous to cerium (P:Ce) from the XPS and EDS spectra, respectively indicate that the phosphate phase is preferentially segregated to the surface of a primarily CeO_2_ backbone (Fig. 1d). This was confirmed using high-resolution transmission electron microscopy (Fig. 1c) and is in-line with the work of Granados et al. on the same material^9^.

Powder X-ray diffraction (PXRD) showed the presence of cubic fluorite-type CeO_2_, as well as the presence of two distinct hexagonal and monoclinic monazite CePO_4_ phases. PXRD spectra of the CeO_2_-CePO_4_ nanorod loaded with Ni showed a Ni(111) peak at a 2θ angle of 49° indicating that the Ni loadings of between 1.91 and 2.38 at.% were not isomorphically substituted into the CeO_2_-CePO_4_ nanorod, but were instead present as metal nanoclusters situated on top of the nanorod to form a heterostructure. The PXRD spectra are available in Supplementary Information**:** Fig. S5.

BET results showed that the surface area of the support increases with the P:Ce ratio in the support (Fig. 1f) The surface area of the nickel-loaded catalyst samples increases drastically from 31.8 m^2^ g^−1^ when the P:Ce ratio is 0, to 74.65 m^2^ g^−1^ for the sample with a support composition of P:Ce = 0.65. This is expected since CePO_4_ is known to have a higher surface area than CeO_2_.

The successful modulation of the surface basicity by controlling the density of phosphate groups on the surface of the photocatalyst support was confirmed via thermogravimetric analysis, which showed an increase in the ability of the catalyst to adsorb the acidic CO_2_ molecule when the Ni-CeO_2_ catalyst is phosphated (see Supplementary Information: Figs. S6, S7). The modulation in surface basicity also resulted in a decrease in Ni particle size (Fig. 1e). Basic supports have been shown to improve metal dispersion and therefore facilitate a smaller metal cluster size because the Lewis basic surface has a high number of non-bonding electrons available to attract and strongly disperse positively charged metal ions^10^ Small nickel clusters have also been observed to mitigate carbon accumulation and subsequent deactivation in thermal dry-reforming catalysts^11^

### Photocatalytic dry reforming using the Ni-CeO_2_-CePO_4_ photocatalyst

**Fig. 2:**
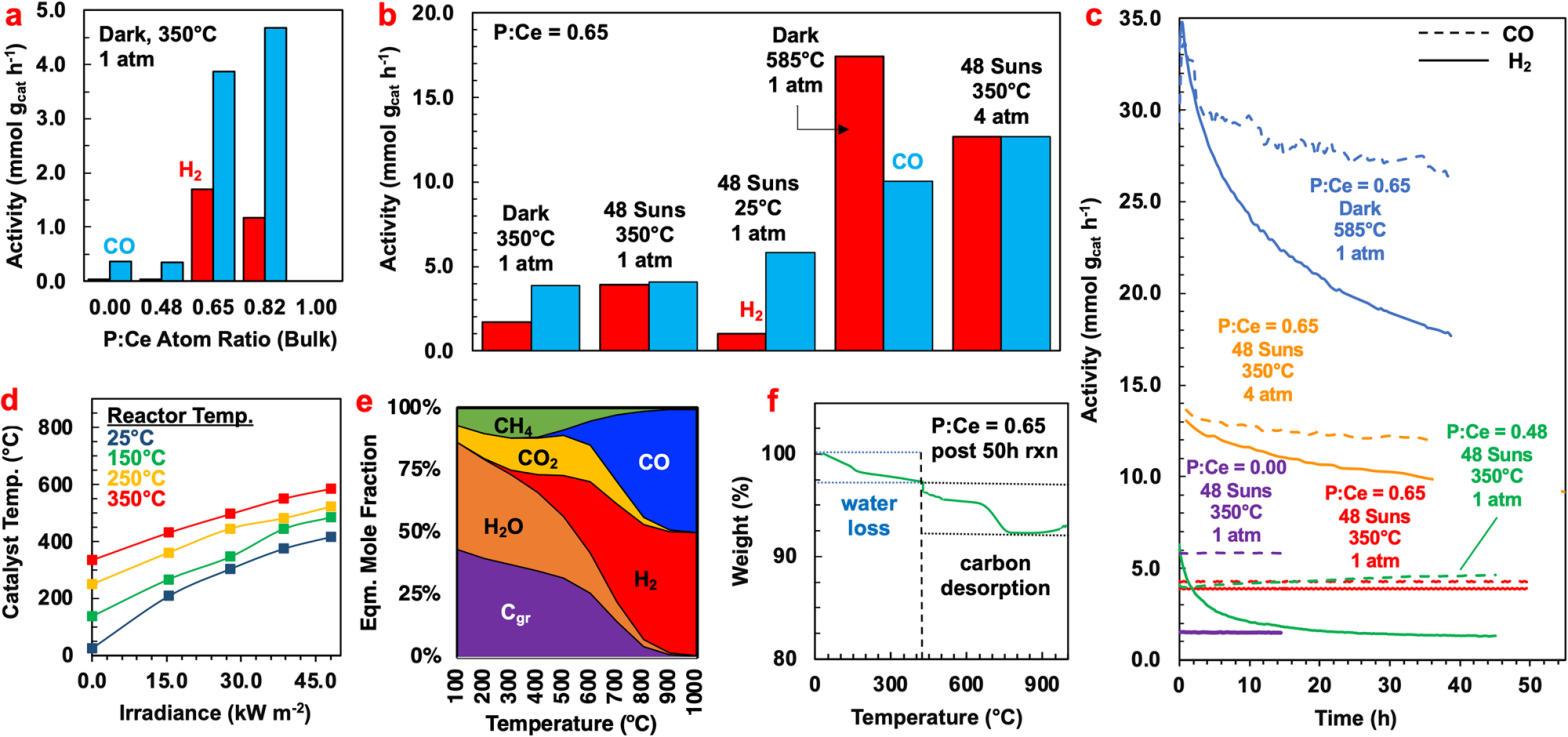
Photocatalytic dry reforming performance of Ni-CeO_2_-CePO_4_ under varying conditions. **a** Catalytic performance of phosphate Ni-CeO2 photocatalyst with varying P:Ce ratios in the support, in the dark. **b** Activities of the Ni-CeO2-CePO4 photocatalyst under varying conditions. **c** Stability of varying Ni-CeO_2_-CePO_4_ photocatalyst samples under varying conditions. **d** Photocatalyst temperature under varying reactor temperatures measured by infrared thermometry. **e** Thermodynamic equilibrium product composition of the dry reforming reaction system at varying temperatures. **f** Thermogravimetric analysis of spent Ni-CeO_2_-CePO_4_ photocatalyst.

Figure 2a shows the activity of the Ni-CeO_2_-CePO_4_ catalysts with varying ratios of P:Ce at a reactor temperature of 350 °C, atmospheric pressure, and no light irradiance. An optimum P:Ce ratio was found to exist between a P:Ce ratio of 0.65 and 0.82. The Ni-CeO_2_-CePO_4_ catalyst with a P:Ce ratio of 0.65 displayed an increase in H_2_ and CO production rates of 56.8 and 10.6 times, respectively, in excess of the unphosphated Ni-CeO_2_ catalyst. Notably, the sample with a P:Ce ratio of 0.48 showed the highest surface area at 82.5 m^2^ g^−1^, although it did not display the enhanced activity that the sample with a P:Ce ratio of 0.65 exhibited, indicating that the enhanced activity is due to an effect beyond the geometric enhancement of the increased surface area.

The lack of activity observed from the sample with a 1:1 ratio of P:Ce in the support is hypothesized to arise from the inability of the CePO_4_ phase to facilitate the switching of the Ce^3+^/Ce^4+^ redox active pair^12^ Monazite or rhabdophane-type CePO_4_ does not facilitate mixed valence Ce^3+^/Ce^4+^ switching, and as a result, the oxygen diffusion capabilities are limited to the CeO_2_ phase of the heterostructure, and the mixed oxidation state of Ce^3+^/Ce^4+^ in the mixed support emerges from the presence of fluorite-phase cerium oxide in the mixed oxide/phosphate heterostructure which agrees with PXRD spectra. The Ni-CeO_2_-CePO_4_ catalyst with a P:Ce ratio of 0.65 exhibited the highest H_2_:CO ratio at these very mild conditions, of 0.44, in comparison to the catalyst with a P:Ce ratio of 0.82, which displayed an H_2_:CO ratio of 0.25 in the product gas mixture and was therefore chosen to be optimized.

To rule out the possibility of the catalyst activity originating from a nickel-phosphide complex on the surface of the catalyst acting as a reaction center rather than the phosphate surface groups, a commercial Ni_2_P powder was tested; however, no activity was observed. This further supports the idea that there exists a synergistic reaction pathway enabled by the mixed-phase CeO_2_-CePO_4_ support that may play an important role in enhancing the photocatalytic activity.

Figure 2b shows the performance of the Ni-CeO_2_-CePO_4_ photocatalyst under varying conditions. Applying 48.1 kW m^−2^ of white light irradiance to the reactor heated to 350 °C was observed to increase the H_2_:CO ratio in the product gas mixture from 0.44 to 0.95 but kept the overall activity of the catalyst roughly the same at 4 mmol g_cat_ h^−1^. The photocatalyst also exhibited excellent stability under these conditions (Fig. 2C). Under 48.1 kW m^−2^ irradiation and in the absence of heating, the H_2_:CO ratio dropped to 0.17. When the pressure in the reactor was raised to 4 atm, the activity of the catalyst increased by 3.1 times; however, the stability under these conditions suffered an activity loss of 0.23% per hour, in comparison to the photocatalyst tested at the same conditions but under 1 atm pressure, which showed negligible activity loss after 50 h.

At a reactor temperature of 350 °C, and under 48.1 kW m^−2^ irradiance, the temperature of the Ni-CeO_2_-CePO_4_ photocatalyst with a P:Ce ratio of 0.65 was measured by infrared thermometry to be approximately 585 °C (Fig. 2d). Under a reactor temperature of 585 °C and no irradiance, the photocatalyst exhibited significantly higher activity than it had under a reactor temperature of 350 °C and 48.1 kW m^−2^ irradiance at 1 atm pressure, however, the H_2_:CO ratio in the product gas mixture was 1.73, indicating the presence of the pyrolysis reaction, and the photocatalyst exhibited an activity loss of 0.11% per hour. Figure 2e shows the equilibrium product composition of a dry reforming reaction system with an equimolar feed of CO_2_ and CH_4_, and at atmospheric pressure, over a range of temperatures, calculated using the Gibbs Free Energy Minimization method, described in detail in other work from our group^13^ As can be seen, there is a strong thermodynamic driving force to produce carbon (C_gr_) below 900 °C, above which the equilibrium mol% of C_gr_ drops to 1.04% (see Supplementary Information: Fig. S1 and Table S1 for equilibrium composition data). This suggests a high propensity for coking under thermal reaction conditions at 585 °C, where the expected mol% of C among reaction products is roughly 26%, and the equilibrium H_2_:CO ratio is 3.38. Although the reaction at hand did not reach equilibrium, the observed H_2_:CO ratios of more than 1 in the product gas mixture are qualitatively indicative of operation in a high-coking regime.

In contrast, the Ni-CeO_2_-CePO_4_ photocatalyst with a P:Ce ratio of 0.65, under 48.1 kW m^−2^ irradiance and a reactor temperature of 350 °C was able to maintain an H_2_:CO ratio of 0.94 over the course of a 50-h stability test. Following 50 h of reaction, the amount of carbon deposited on the surface of the catalyst was quantified using thermogravimetric analysis (TGA), in which the photocatalyst was heated from 23 to 1000 °C at a rate of 10 °C min^−1^, while dry air flowed over the sample at a rate of 3.5 L min^−1^. Weight loss below 426 °C was assumed to be due to water loss and amounted to 2.7 wt% (Fig. 2F). The remaining weight loss above 426 °C was attributed to carbon desorption, which was measured to be 4.2 wt%, which, over the course of the 50-h reaction, is the equivalent of a carbon deposition rate of 1.5 × 10^−4^ mmol g_cat_^−1^ h^−1^, ~3.8% of the H_2_ and CO production rates.

This discrepancy in activity between the purely thermal and photo-assisted results suggests that the photocatalytic activity observed may not be purely due to a photothermal effect. The type of response displayed by the catalytic activity in response to increasing irradiance has been noted to be indicative of the underlying photocatalytic pathway at play^14–16^ An exponential increase in photocatalytic activity with respect to increased radiative flux is likely due to photothermal effects since an increase in reaction temperature is known to produce this Arrhenius-type kinetics (*k* = *A*^*−Ea/RT*^). In contrast, if an incident photon does not increase the temperature experienced by the system, it is possible that the work done to accomplish the surface chemical reaction is done by the photochemically generated electron-hole pair. In this scenario, since the absorption of a photon destined to do photochemistry can be assumed to create one electron-hole pair, or a single value of one of the other excited state modes previously described, a more linear dependence in reaction rate to a radiant flux of photons absorbed by the catalyst is expected. As photon-induced pathways become saturated at a high photon flux, the reaction rate may taper off, resembling a root law dependency. A combination of the two pathways may appear as a supra-linear dependency of the reaction rate on radiative flux.

To probe the nature of the light-induced photocatalytic mechanism, the photocatalytic activity under specific wavelengths of light was measured at varying light intensities, using four LED lamps with light spectra confined to a specific segment of the UV- and visible light spectra (UV, blue, red, and green). For these tests, three of the four LED lamps were held at 100% power while the fourth LED lamp, representing the light wavelength under examination, was then varied over a range of irradiances (See Supplementary Information: Fig. S8). This allowed for the observation of the change in photocatalytic activity resulting from an increase in irradiation from that specific wavelength range of light, while considering the interactions that may exist between light-induced pathways, since electronic excitations leading to either photochemical activity or local heating on the catalyst surface occur simultaneously and act synergistically such that the measured activity is likely not a strict superposition of the two effects, and the two effects may not be definitively and quantitatively decoupled, which is an existing issue in the field.

**Fig. 3:**
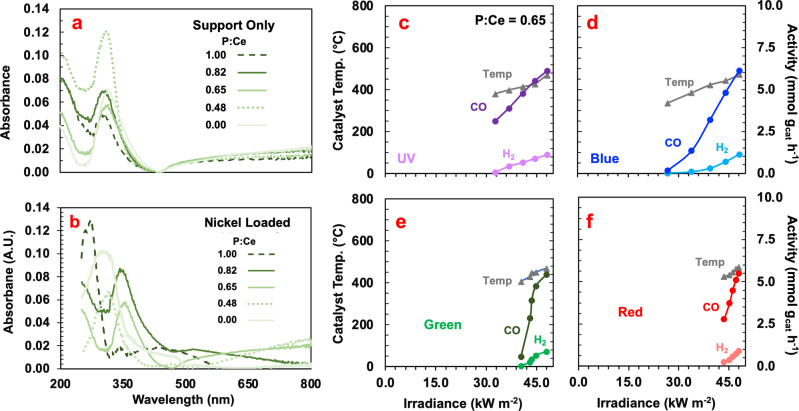
Wavelength dependence of the observed photocatalytic activity. **a**, **b** Optical absorption spectra for the CeO_2_-CePO_4_ support and nickel-loaded samples with varying P:Ce ratio. **c**–**f** Wavelength-specific activities and catalyst temperatures of Ni-CeO_2_-CePO_4_ photocatalyst with a P:Ce ratio of 0.65, a reactor temperature of 350 °C.

The response in photocatalytic activity with increased irradiance was observed to follow different trends depending on the wavelength being varied, providing insight into the potential photochemical or photothermal nature of the photocatalyst’s response to that wavelength of light. The results shown in Fig. 3c–f indicate that blue light (425–515 nm) and green light (475–600 nm) have the largest impact on the photocatalytic activity, as when they are at 0%, the photocatalytic rate is low, despite the other lamps being held at 100%. In contrast, when the red (575–650 nm) and UV (350–425 nm) lamps are off, the photocatalytic rate is already significant. The corresponding catalyst temperature was observed to increase by the largest amount when the blue light was varied, followed by the UV, green, and red lights. This differed from the increase in rate observed with each wavelength, which increased most as the blue light intensity was increased, followed by the green, UV, then red, further indicating the presence of a mixed photothermal-photochemical activity.

Modulation in the intensity of the UV light produced a linear change in the observed reaction rate, whereas changing the intensity of the blue light produced an exponential response, the change in the green light produced a concave shape, and the change in red light resulted in a linear response, indicating that the nature of the photocatalytic pathway may differ depending on the energy of the photon absorbed. While it is common in the academic literature to ascribe photocatalytic activity to one of these pathways, it is observed that each is present and contributes to the photocatalytic activity uniquely.

The increase in blue light intensity resulted in an exponential-type response, indicative of a dominant photothermal effect in this region, whereas the response to illumination with UV light appears to be photochemical. This is likely caused by absorption by the CeO_2_-CePO_4_ support, as both components have been reported to absorb in the 280 to 440 nm range (Fig. 3a)^17–20^ The CeO_2_ absorption peak is attributed to an electron transfer from the O^2−^ 2p orbital to the Ce^4+^ conduction band, while the CePO_4_ absorption peak represents the absorption resulting from the ligand-to-metal charge transfer from the phosphate group to the Ce^20^ A Tauc plot was used to determine the apparent bandgap of the photocatalyst support^21^ It was determined that the addition of phosphate into the photocatalyst support, reduced the optical bandgap from 2.91 to 2.53 eV, potentially increasing the proportion of incident photons that are able to be absorbed.

Because the CeO_2_-CePO_4_ support does not absorb in the green or red regions (Fig. 3a), the activity resulting from an increase in green or red-light irradiation was attributed to absorbance by nickel, the addition of which resulted in a second absorption peak (Fig. 3b). This broad peak in the green region has been attributed to the excitation of the nickel plasmon resonance, which can have several effects on the observed photocatalytic rate^22–24^ It is unlikely that the observed increase in rate is due to hot-carrier processes, due to their more likely decay at higher temperatures. Alternatively, thermal decay modes can heat the metal oxide semiconductor locally, resulting in an increased effective reaction temperature. Further, the well-documented “antenna effect” may be at play wherein a metal nanoparticle coupled to a metal oxide support can allow for light collection from an area that is larger^25^ than the geometric area of the metal nanoparticle, which has been shown to enhance the metal oxide’s light absorption in the visible part of the spectrum via charge-transfer induced modification of the metal oxides optoelectronic properties^26^ The observed saturation in the response of the photocatalytic activity to an increase in green light irradiance can potentially be attributed to this latter effect since the enhancement in light absorption is finitely associated with the size of the metal nanoparticle and therefore can reach a saturation point where additional incident photons would not be absorbed.

**Fig. 4:**
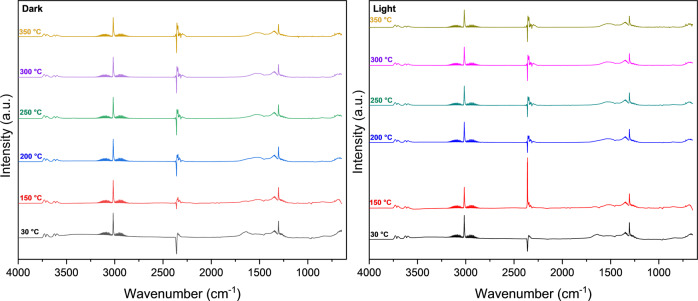
In situ DRIFTS spectra for dry reforming reaction. In situ DRIFTS spectra of the dry reforming reaction using the Ni-CeO_2_-CePO_4_ photocatalyst with a P:Ce ratio of 0.65 taken under dark and light conditions at various temperatures.

To further investigate whether the photocatalytic reaction mechanism at hand is driven by photothermal or photochemical means, in situ diffuse reflectance infrared Fourier transform spectroscopy (DRIFTS) was performed on the optimum catalyst composition under dark and light conditions at varying reactor temperatures to observe whether the reaction pathways are potentially altered from the thermal pathway when the dry reforming reaction is carried out photocatalytically. The spectra, shown in Fig. 4 (and the Supplementary Information: Fig. S9), were analyzed using Sigma-Aldrich’s IR Spectrum Table Database^27^ Peaks corresponding to the symmetric phosphate groups (1042 cm^−1^), hydroxyl groups (3854 cm^−1^), carbonyls species (1830−2041 cm^−1^), chemisorbed H_x_CO_y_ species (1329─1803 cm^−1^), carbon dioxide (2349 cm^−1^), and C-H alkane bond stretching (3000−2840 cm^−1^), C-O bond stretching^28^ (1240 cm^−1^), and C-H alkane bond bending (1304 cm^−1^) were observed.

The peak near 3016 cm^−1^ is due to the C–H stretching of CH_4_, whereas the peak around 1304 cm^−1^ is assigned to the C–H bending of CH_3_ and CH_2_, indicative of the well-documented step-wise decomposition of CH_4_ on Ni dry reforming catalysts, which can lead to carbon accumulation if the CH_x_ decomposition is allowed to proceed to an x = 0 state^29^ Correspondingly, the O-H stretching groups suggest that the abstracted H atoms bind to O sites on the catalyst support. The bands at 1680, 1417, and 1209 cm^−1^ were attributed to hydrogen carbonates vibrations, and the peak at 1642 cm^−1^ was assigned to bridged carbonates. The broadband (1472 and 1530 cm^−1^) was attributed to mono-carbonates. Under dark conditions, the weakly bound bridged carbonates are being transformed into mono-carbonates and new bands (1583, 1371, and 1343 cm^−1^) attributed to formate’s formation^30^ These two key intermediates monodentate or bidentate carbonates indicating a good adsorption and activation capacity of the catalyst for CO_2_.

Under illumination, the C-H bending peaks (1304 cm^−1^) are intensified, as the ones at 1583 and 1371 cm^−1^, attributed to the vibration of monodentate formate (υ_asym_(COO)), while the peaks related to hydrogen carbonate species (1680, 1417, or 1209 cm^−1^) are decreased. Additionally, under illumination, the appearance of the carbonyl species on the Ni surface, was identified at 2041 cm^−1^ (monocarbonyl), 1927 cm^−1^ (bridged carbonyl), and 1830 cm_−1_ (twofold bridged carbonyl). This indicates that carbonyls take part in the process of the dry reforming reaction under illumination, a potentially light-specific pathway. On the other hand, the CO_2_ stretching peak near 2354 cm^−1^ was more intense under dark than under light, indicating a higher conversion under light conditions.

## Discussion

In comparison to the other photocatalytic dry reforming systems presented in the academic literature, the Ni-CeO_2_-CePO_4_ photocatalyst described herein exhibited good stability. The deactivation rate ranged from 0.0 to 0.23% per hour depending on the conditions used (Fig. 2c), which is on the low end of the deactivation rates reported in the literature examples described in the introduction, which report deactivation rates between 0.0 and 7.5% per hour (see Supplementary Information: Table S2).

Comparison with efficiency values present in the photocatalytic dry reforming literature is challenging as few of the photocatalytic dry reforming papers in the academic literature report a quantum efficiency, and each that does, does so in a unique manner. Shoji et al. reported a quantum efficiency of 5.9%, calculated as the electron number for the reaction per absorbed photon. Han et al., report an energy efficiency based on purely photothermal performance and assumed the presence of only the dry reforming and reverse water gas shift reactions and reported a value of 57.8%, albeit at a very high temperature of 650 °C, making it difficult to compare mechanisms^6,7,31^ Zhou et al. used a similar method and reported an efficiency of 15% although the performance of their catalyst is attributed to hot-carrier-driven processes, and is technically distinct from a purely photothermal operation. Zhang et al.^7^ reported a quantum efficiency of 11.1% based on a “light-to-fuel” efficiency based on the production or consumption rates of H_2_, CO, CO_2_, and CH_4_, and the heats of combustion or higher heating values of H_2_, CO, and CH_4_.

Further to the challenge of comparing photocatalysts, some reports normalize their rates and efficiency calculations to the mol% of precious metal nanoparticles on the metal oxide support, which can make comparisons between catalyst activities inconsistent. For example, Zhou et al. normalize their rates, turnover frequencies, and efficiencies to the Ru component of their catalyst, which makes up 0.1% of the photocatalyst composition^5^ and assumes that the reaction takes place solely on the CuRu alloy that is situated on an MgO-Al_2_O_3_ support, two components of which have been reported to elicit strong metal-support interactions in the dry reforming system and therefore likely have a catalytic purpose and should not be ignored.

Because of the hybrid nature of the Ni-CeO_2_-CePO_4_ photocatalytic dry reforming system presented herein, both apparent quantum and energy efficiencies were calculated for comparison to the literature examples described above. An apparent quantum efficiency^32^ was calculated as the molar production rate of H_2_ or CO (mol H_2_ h^−1^) per molar photon flux (mol h^−1^), and the energy efficiency was calculated as $\eta \,=\,{r}_{{{{{{{\mathrm{H}}}}}}}_{2}}\triangle {H}_{{{{{{{\mathrm{DRM}}}}}}}}^{^\circ }/\phi$ where η is the energy efficiency, ${r}_{{H}_{2}}$ is the molar production rate of H_2_, $\triangle {H}_{{{{{{{\mathrm{DRM}}}}}}}}^{^\circ }$ is the enthalpy of reaction for the main dry reforming reaction, and $\phi$ is the photon flux. The optimal conditions above that produced stable operation were observed to have a quantum efficiency of 0.59 and 0.62% for H_2_ and CO production, respectively, and an energy efficiency of 0.7%, which is higher than those reported by Zhou and Shoji after their efficiencies have been corrected to reflect the true catalyst mass. It should be noted that these energy efficiency values also omit the energy spent heating the reactor and gases.

Apparent turnover frequencies (TOF_app_) for the literature examples described above were calculated as the photocatalytic rate of production per unit surface area of the photocatalyst, for those that reported both of those values (see Supplementary Information: Table S2). From this perspective, the highest values reported were by Zhang and Mao and resulted in apparent turnover frequencies of 7.73 × 10^−3^ and 2.78 × 10^−3^ mol m^−2^ h^−1^. These values were comparable to the Ni-CeO_2_-CePO_4_ photocatalyst when run at a higher pressure of 4 atm (TOF_app_ = 1.08 × 10^−3^ mol m^−2^ h^−1^) but were not surpassed by the stable 1 atm operation, which displayed a TOF_app_ of 5.23 × 10^−5^ mol m^−2^ h^−1^, although it displayed comparable efficiencies.

## Methods

### Catalyst synthesis and characterization

The Ni-CeO_2_-CePO_4_ photocatalyst was produced by first synthesizing a mixed CeO_2_-CePO_4_ support using an aqueous room temperature synthesis followed by the addition of Ni to the structure using the incipient wetness technique. An amount of Ce(NO_3_)_3_·H_2_O (99% pure, trace metals basis) was dissolved into deionized water, and a stoichiometrically appropriate amount of H_3_PO_4_ (85 wt%) was added to the solution, at an amount that reflects the percentage of the dissolved cerium that is intended to form CePO_4_. Ammonia was then added dropwise until the pH of the solution reached 9. The mixture was then stirred at room temperature for 2 h and aged at room temperature for an additional 24 h. The resulting product solution was then centrifuged and washed with deionized water repeatedly until the pH of the supernatant liquid reached 7. The separated powder samples were then dried at 80 °C in air, and then calcined for 3 h at 450 °C. Ni was loaded onto the support via an aqueous Ni(NO_3_)_2_ solution using the incipient wetness technique, followed by calcination for 3 h at 450 °C. The samples were then reduced at 650 °C under a 10% H_2_/Ar mixture for 3 h.

Physical characterization of the catalyst material was carried out as follows. An FEI Quanta FEG 250 scanning electron microscope equipped with STEM and EDAX detectors was used to determine the structure and composition of the resulting Ni-CeO_2_-CePO_4_ catalysts with varying support compositions. The morphology of the catalyst before and after reduction was examined using transmission electron microscopy (TEM, Hitachi HT7700) under 100 kV. An aqueous dispersion of the catalyst was applied to form var-coated 200 mesh copper TEM grids (Electron Microscopy Sciences). X-ray diffraction was carried out on a Bruker D2-Phaser X-ray diffractometer using Cu Kα radiation generated at 30 kV. The BET surface area of the samples was measured using a FlowSorb analyzer from Micromeritics. Optical absorbance was measured using a Lambda 1050 UV-vis spectrometer equipped with a 150 mm integrating sphere. Catalyst powder samples were loaded onto borosilicate filter paper for measurement. CO_2_ Uptake (adsorption) measurements were performed in a TGA discovery instrument. Initially, each sample was preheated in a CO_2_ (20 mL min^−1^) atmosphere at 120 °C to remove any dust, moisture, and impurities from the sample surface for 30 min, followed by a ramp of 30 °C min^−1^ up to 400 °C. The sample was then equilibrated until 60 °C and held isothermally for 30 min. During this step, the CO_2_ is adsorbed on the surface of the sample and was measured by a mass weight difference. Several cycles of the above procedure were performed to evaluate the stability of the sample under CO_2_ conditions.

Quantitative analysis of carbon on the spent catalysts was performed using thermogravimetric analysis. The weight loss was measured by loading the mass in µg of the sample. The sample was oxidized by increasing the temperature to 1000 °C at 10 °C/min under a flow of dry air (3.5 L min^−1^). The amount of coke is calculated from the following equation: (Amount of coke) = (sample weight at 23 °C)- (sample weight at 1000 °C)/(sample weight at 23 °C)

In situ diffuse reflectance infrared Fourier transform spectroscopy (DRIFTS) spectrums were collected on a Thermo Scientific iS50 series Fourier transform infrared (FT‐IR) equipped with a liquid nitrogen‐cooled MCT detector, to study the nature of intermediate species formed during the dry reforming reaction over the Ni-CeO_2_-CePO_4_ catalyst under dark and light-assisted reaction conditions (10 W white LED irradiation). The catalyst was placed into the holder of a Harrick reaction chamber and pre-reduced at 400 °C under 5% H_2_/Ar flow of 20 mL/min for 30 min, then purged in 20 mL/min Ar gas for 1 h to remove the residual H_2_ from the cell and cooled down to different temperatures (30–350 °C). The background spectrum was recorded in Ar at different temperatures. A dry reforming reaction was performed in 20 mL/min of gas mixture (10 sccm Ar + 5 sccm CO_2_ + 5 sccm CH_4_) at different temperatures. Each measurement was recorded after 30 min in isothermal conditions and followed by purging in Ar for 30 min to remove the excess water molecules. Spectra were recorded from 4000 to 649 cm^−1^ with a step of 1 cm^−1^.

To investigate the magnitude of heating produced by the illumination of the catalyst with light, infrared thermography using a forward-looking infrared camera (FLIR) was carried out to estimate the global temperature of the catalyst bed when illuminated under different light intensities. A thermal imaging camera (Therma- CAM EC320 FLIR) was directed at the sample through the reactor window while it was being simultaneously irradiated with light. For these measurements, the catalyst sample was placed in the reactor at the focal point of the light, the same approximate distance from the light source as within the reactor. The LEDs were focused on the sample. Then, the FLIR camera is pointed at the sample to measure the radiated heat caused by the concentrated light.

### Catalytic activity measurements

The laboratory-scale photoreactor used to conduct the experiments presented in this work was comprised of a Harrick HTC-3 High-Temperature Cell fitted with a fused silica window. Pressure in the reactor was controlled by tightening or loosening an in-line diaphragm and monitoring the pressure using an Omega pressure transducer. The catalyst sample is placed on a steel mesh, underneath which is an electrical resistance heater. The temperature setting of the electrical resistance heater is noted as the “reactor temperature” and does not necessarily represent the catalyst temperature under reaction conditions and when illuminated with light. The catalyst sample was illuminated by an array of four Prizmatix LEDs (UV (UHP-T-365-MP), Blue (UHP-T−460-DI), Green (UHP-T−520-DI), and Red (UHP- 625-DI)), as well as a beam combiner. The power output of each lamp can be modulated independently. The power output for each LED was measured using a power meter at the same position as the sample within the reactor. The irradiance produced by the Prizmatix LEDs was measured using a light meter. For the present examples, a nominal flow rate of 10 mL min^−1^ was used, with the reactant feed consisting of equal parts CO_2_ and CH_4_ (2.5 mL min^−1^ each) with a balance of 5 mL min^−1^ Ar. The evolution of reaction products was measured using an in-line SRI 8610c gas chromatography.

## Supplementary information

Supplementary Information

## Data Availability

The data that support the findings of this study are available from the corresponding author upon reasonable request.
