# Author Correction: Enhanced hybrid photocatalytic dry reforming using a phosphated Ni-CeO_2_ nanorod heterostructure

**DOI:** 10.1038/s41467-023-37493-x

**Published:** 2023-03-30

**Authors:** Alexandra Tavasoli, Abdelaziz Gouda, Till Zähringer, Young Feng Li, Humayra Quaid, Camilo J. Viasus Perez, Rui Song, Mohini Sain, Geoffrey Ozin

**Affiliations:** 1 Department of Chemistry, University of Toronto, 80 St. George Street, M5S 3H6 Toronto, Canada; 2 Department of Materials Science & Engineering, University of Toronto, 184 College St., M5S 3E4 Toronto, Canada; 3 Department of Mechanical and Industrial Engineering, University of Toronto, 5 King’s College Rd, Toronto, ON M5S 3G8 Canada

**Keywords:** Photocatalysis, Heterogeneous catalysis

Correction to: *Nature Communications* 10.1038/s41467-023-36982-3, published online 15 March 2023

In this article the author name Till Zähringer was incorrectly written as Till Zahringer. The original article has been corrected.

