# Supplementary material for: Enhanced hybrid photocatalytic dry reforming using a phosphated Ni-CeO2 nanorod heterostructure
Source: Nat Commun. 2023 Mar 15;14:1435. doi: 10.1038/s41467-023-36982-3 (PMC10015045; doi:10.1038/s41467-023-36982-3)
Supplement: Supplementary file 1 — Supplementary Information [file 41467_2023_36982_MOESM1_ESM.pdf]

SUPPLEMENTARY INFORMATION

FOR

"ENHANCED PHOTOCATALYTIC DRY REFORMING THROUGH SURFACE BASICITY MODULATION USING A PHOSPHATED NI-CEO$_2$ NANOROD HETEROSTRUCTURE"

Alexandra Tavasoli[1,2,§], Abdelaziz Gouda[1,3,§], Till Zahringer[1], Young Feng Li[1], Humayra Quaid[2], Camilo J. Viasus Perez[1], Rui Song[1], Mohini Sain[3], Geoffrey Ozin[1,*]

[1] Department of Chemistry, University of Toronto, 80 St. George Street, Toronto, Canada, M5S 3H6
[2] Department of Materials Science & Engineering, University of Toronto, 184 College St., Toronto, Canada, M5S 3E4
[3] Department of Mechanical and Industrial Engineering, University of Toronto, 5 King's College Rd, Toronto, ON M5S 3G8, Canada
[§] These authors contributed equally
* Correspondence should be forwarded to Geoffrey Ozin at g.ozin@utoronto.ca

Free energies of dry reforming network reactions

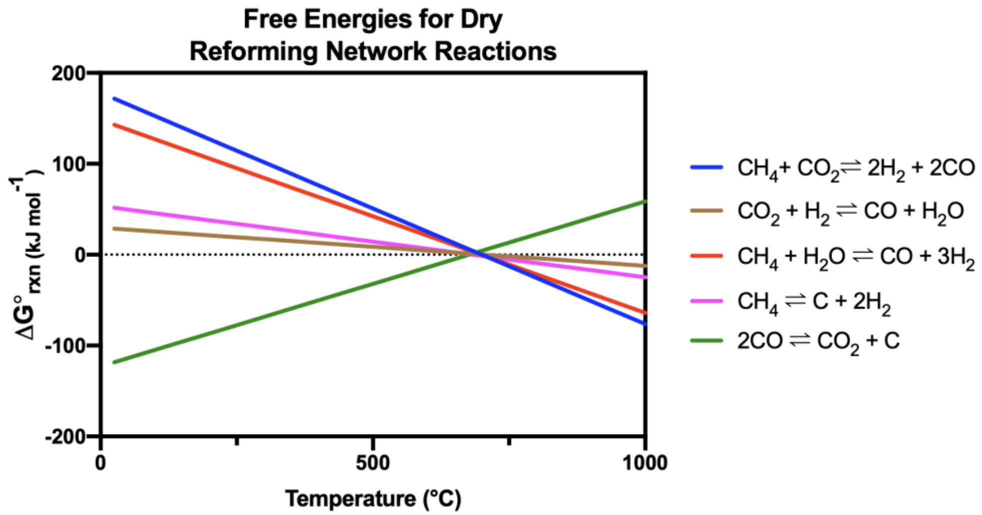

Figure S1. Free energies calculations of dry reforming network reactions with temperature.

Equilibrium Product Compositions

Table S1. Equilibrium compositions were calculated using a Gibbs Free Energy Minimization method described in detail in our other publication.[1]

T (°C)	100	200	300	400	500	600	700	800	900	1000
CH_4	7%	10%	12%	12%	9%	5%	2%	1%	0%	0%
CO	0%	0%	0%	0%	2%	10%	27%	43%	48%	50%
CO_2	7%	10%	13%	15%	16%	15%	9%	3%	1%	0%
H_2	0%	0%	2%	7%	17%	29%	40%	46%	49%	49%
H_2O	43%	40%	36%	32%	25%	16%	8%	3%	1%	0%
$C_{(GR)}$	43%	40%	37%	35%	32%	26%	14%	4%	1%	0%

Performance of dry reforming photocatalysts reported in the academic literature, and comparison to Ni-CeO_2-$CePO_4$ catalyst

Han *et al.*[2] demonstrated photocatalytic DRM using a Pt-TiO_2-SiO_2 photocatalyst and was able to achieve an H_2:CO ratio of 0.04 using 1 kW m^{-2} irradiance (equivalent to "1 sun" of irradiance) and a reactor temperature of 650°C. Han reported a quantum efficiency of 57.8% at these conditions, and the catalyst activity was shown to decline by approximately 2.17% per hour. Zhou[3] demonstrated a CuRu-MgO-Al_2O_3 photocatalyst that achieved a 1:1 ratio of H_2:CO in the product gas mixture at atmospheric pressure using a relatively high light intensity of 192 kW m^{-2} but in the absence of reactor heating. The catalyst was shown to be very stable, losing only approximately 0.22% of its activity per hour. Due to the claimed photothermal nature of Zhou's photocatalyst activity, an energy efficiency of 15% was reported on the basis of incident light energy converted to chemical energy in the form of reaction enthalpy. Shoji *et al.* [4] studied an Rh-$SrTiO_3$ photocatalyst which was shown to achieve a 1:1 ratio of H_2:CO in the product gas mixture at a reactor temperature of 200°C and catalyst irradiation of 1.5 kW m^{-2}. Their catalyst showed a deactivation of 4% per hour, and they reported a quantum efficiency of 5.9%. Pan *et al.*[8] studied a Pt-SiO_2-CeO_2 photocatalyst, and was able to achieve an H_2:CO ratio in the product gas mixture of 0.58 under 30 kW m^{-2} irradiance and a reactor temperature of 180°C, at atmospheric pressure. Their system showed little deactivation over a 30-hour period. Pan *et al.*[5] also studied a Pt-ZnO-CeO_2-MgO photocatalyst and achieved an H_2:CO ratio of 0.69 using a reactor temperature of 600°C and 30 suns of illumination at atmospheric pressure. This catalyst also showed little deactivation over a 30-hour test period. Similarly, Mao *et al.*[6] studied a Pt-CeO_2 photocatalyst and achieved an H_2:CO ratio of 0.34 using an irradiance of 1.00 kW $m^{-2,}$ and a reactor temperature of 500°C. Zhang *et al.*[7] achieved a 1:1 ratio of H_2:CO in the product gas mixture under 363.4 kW m^{-2} and no reactor heating using a Ni-CeO_2 catalyst. They reported an energy efficiency of 11.1%, and a catalyst deactivation rate of approximately 7.5% per hour. Similarly, Lorber *et al.*[8] studied Ni-CeO_2 and achieved an H_2:CO of 0.4 in the product gas mixture using a reactor temperature of 400°C and irradiation of 7.9 kW m^{-2}. A catalyst deactivation rate of 0.71% per hour was reported.

Table S2. Performance of dry reforming photocatalysts reported in the academic literature compared to Ni-CeO_2-$CePO_4$ catalyst reported in this work:

Reference	Catalyst Composition	Surface Area (m^2 g^{-1})	Reactor Temp. (°C)	Illumination (kW m^{-2})	Pressure (atm)	r_{H2} (mol g_{cat} h^{-1})	CO (mol g_{cat} h^{-1})	H_2:CO Ratio	Deactivation Rate (% h^{-1})	TOF_{app} H_2 (mol m^{-2} h^{-1})	TOF_{app} CO (mol m^{-2} h^{-1})	Quantum/Energy Efficiency (%)
Lorber et al., App. Cat B: Env (301) 120745 (2022)	Ni/CeO2	84.0	RT	7.9	NR	NR	NR	--	0.71%	--	--	NR
Shoji et al., Nature Catalysis (3) pp. 148-153 (2022)	Rh/CeO2	NR	RT	5.6	NR	NR	NR	--	NR	--	--	NR
Zhou et al., Nature Energy (5) pp. 61-70 (2020)	CuRu/MgO-Al2O3	NR	RT	160	1	0.396	0.396	1.00	0.22%	--	--	NR
Zhang et al., App. Cat. B: Env (239) pp.555-564 (2021)	Ni/CeO2	50.7	RT	363.4	NR	0.392	0.376	1.04	7.50%	7.73E-03	7.42E-03	11.1 (EE)
Pan et al., App. Cat. B: Env. (260) 118189 (2020)	MgO/Pt/Zn-CeO2	NR	600	30	NR	0.356	0.516	0.69	0.00%	--	--	NR
Mao et al., Green Chemistry (20) pp. 2857-2869 (2018)	Pt/CeO2	123.0	500	1	NR	0.342	0.360	0.95	0.16%	2.78E-03	2.93E-03	NR
Pan et al., Chem. Cat. Chem. (10) pp. 940-945 (2018)	Pt/Si-CeO2	NR	180	30	NR	0.090	0.154	0.58	0.00%	--	--	NR
Tavasoli et al., Present Work (P:Ce = 0.65, 350C, 4 atm, 48 Suns)	Ni/CeO2/CePO4	74.6	350	48.1	4	0.081	0.080	1.01	0.23%	1.08E-03	1.07E-03	NR
Shoji et al., Nature Catalysis (3) pp. 148-153 (2020)	Rh/SrTiO3	NR	200	1.5	1	0.054	0.054	1	4.00%	--	--	5.9 (QE)
Han et al., ACS Catalysis (6) pp. 494-497 (2015)	Pt/TiO2-SiO2	48.0	650	1	NR	0.013	0.370	0.04	2.17%	2.71E-04	7.71E-03	57.8 (QE)
Tavasoli et al., Present Work (P:Ce = 0.65, 25C, 1 atm, 48 Suns)	Ni/CeO2/CePO4	74.6	25	48.1	1	0.009	0.039	0.23	--	1.16E-04	5.17E-04	0.1% / 0.7% (QE/EE)
Tavasoli et al., Present Work (P:Ce = 0.65, 350C, 1 atm, 48 Suns)	Ni/CeO2/CePO4	74.6	350	48.1	1	0.004	0.004	0.91	Negligible	5.23E-05	5.75E-05	NR
Tavasoli et al., Present Work (P:Ce = 0.65; 350C, 1 atm, Dark)	Ni/CeO2/CePO4	74.6	350	0	1	0.002	0.004	0.44	--	2.28E-05	5.18E-05	NR
Tavasoli et al., Present Work (P:Ce = 0.65, 585C, 1 atm, Dark)	Ni/CeO2/CePO4	74.6	585	0	1	0.000	0.029	0.01	--	5.66E-06	3.93E-04	NR

TEM

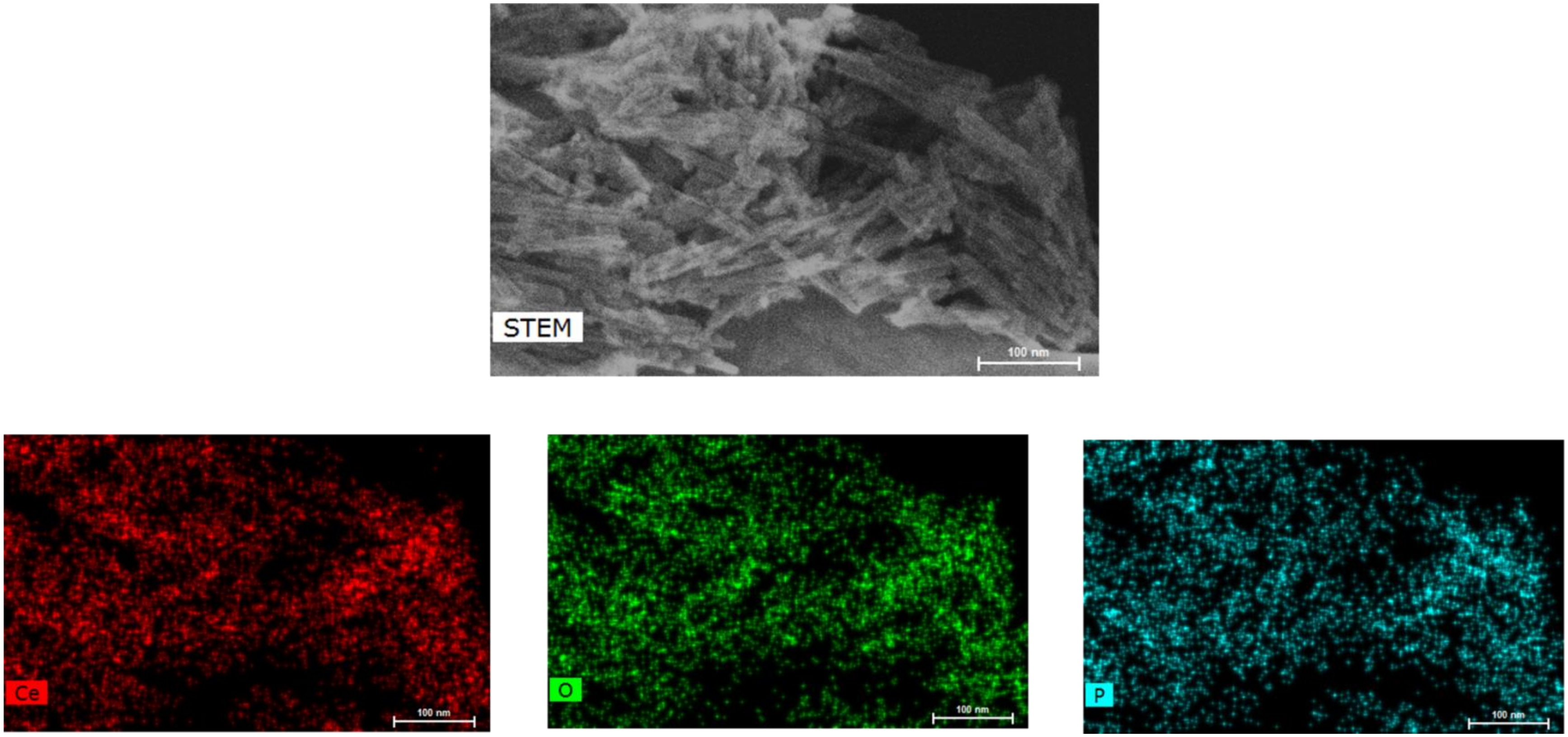

Figure S2. STEM images with EDS mapping for the CeO_2-$CePO_4$ photocatalyst support.

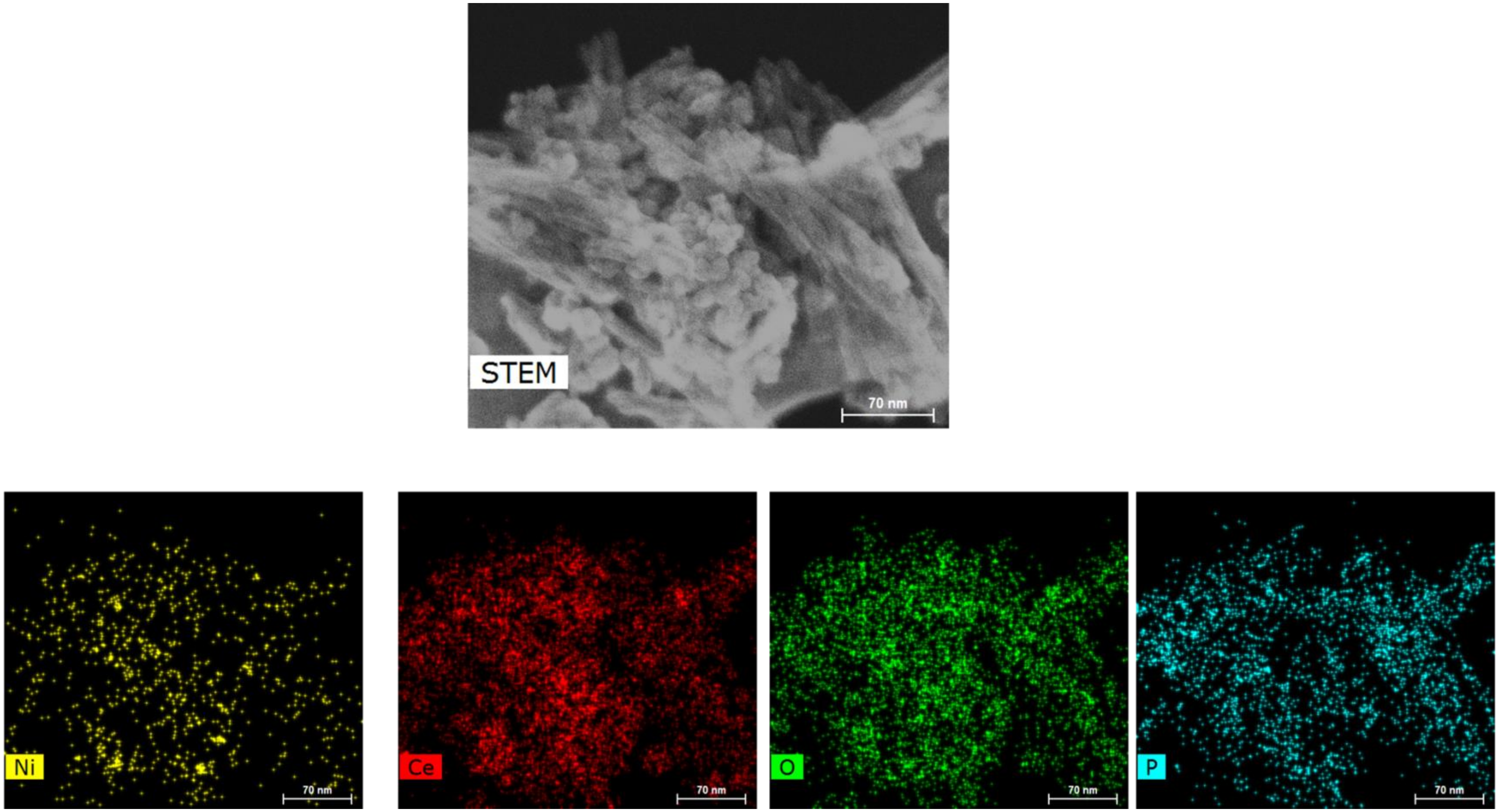

Figure S3. STEM images with EDS mapping for the Ni-CeO_2-$CePO_4$ photocatalyst.

Nominal and measured bulk compositions of Ni-CeO_2-$CePO_4$ photocatalyst samples measured via EDS

Table S3. Nominal and measured bulk compositions of Ni-CeO_2-$CePO_4$ photocatalyst samples measured via EDS

Nominal Catalyst Composition	P:Ce Atom Ratio (EDX)	Ni Content (at%)
10% Ni – P:Ce = 0.00	0 ± 1.60	2.37 ± 32.38
10% Ni – P:Ce = 0.25	0.48 ± 1.83	2.20 ± 35.35
10% Ni – P:Ce = 0.50	0.65 ± 0.64	1.91 ± 36.40
10% Ni – P:Ce = 0.75	0.82 ± 1.07	2.29 ± 36.76
10% Ni – P:Ce = 1.00	1.01 ± 1.13	2.38 ± 25.17

XPS spectra

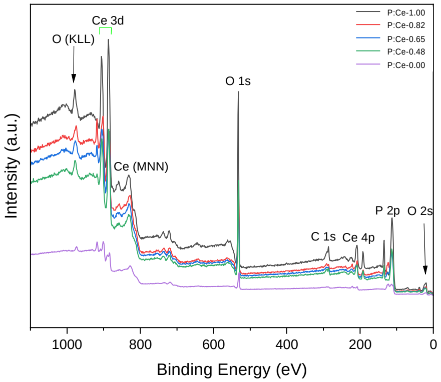

	BE (eV)	P:Ce-0.00	P:Ce-0.48	P:Ce-0.65	P:Ce-0.82	P:Ce-1.00
**O 1s**	523.08	69.43 at%	56.44 at%	60.99 at%	63.66 at%	51.40 at%
**C 1s**	286.08	24.12 at%	21.94 at%	20.01 at%	20.44 at%	23.52 at%
**Ce 3d**	886.08	6.45 at%	5.83 at%	6.05 at%	5.73 at%	4.84 at%
**P 2p**	134.08	0.00 at%	15.80 at%	10.16 at%	10.16 at%	20.25 at%

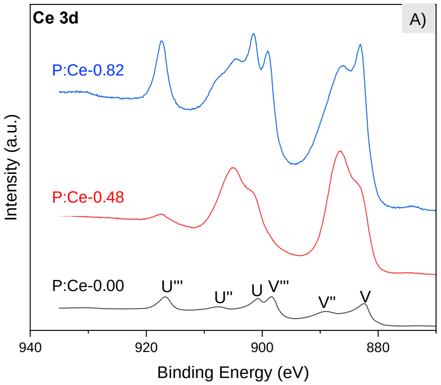

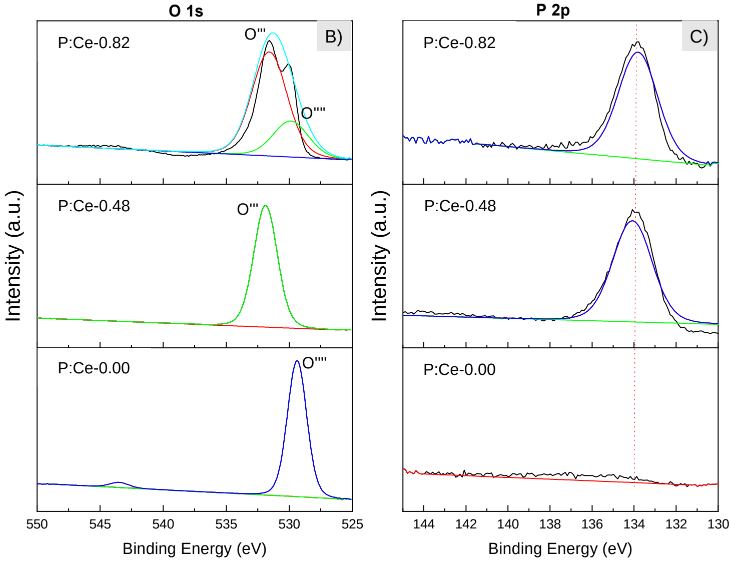

Figure S4. XPS survey scan with Ce 3d, O 1s and P 2p high resolution spectra and elements quantification for the Ni-CeO_2-$CePO_4$ photocatalyst of different P:Ce ratios

PXRD spectra

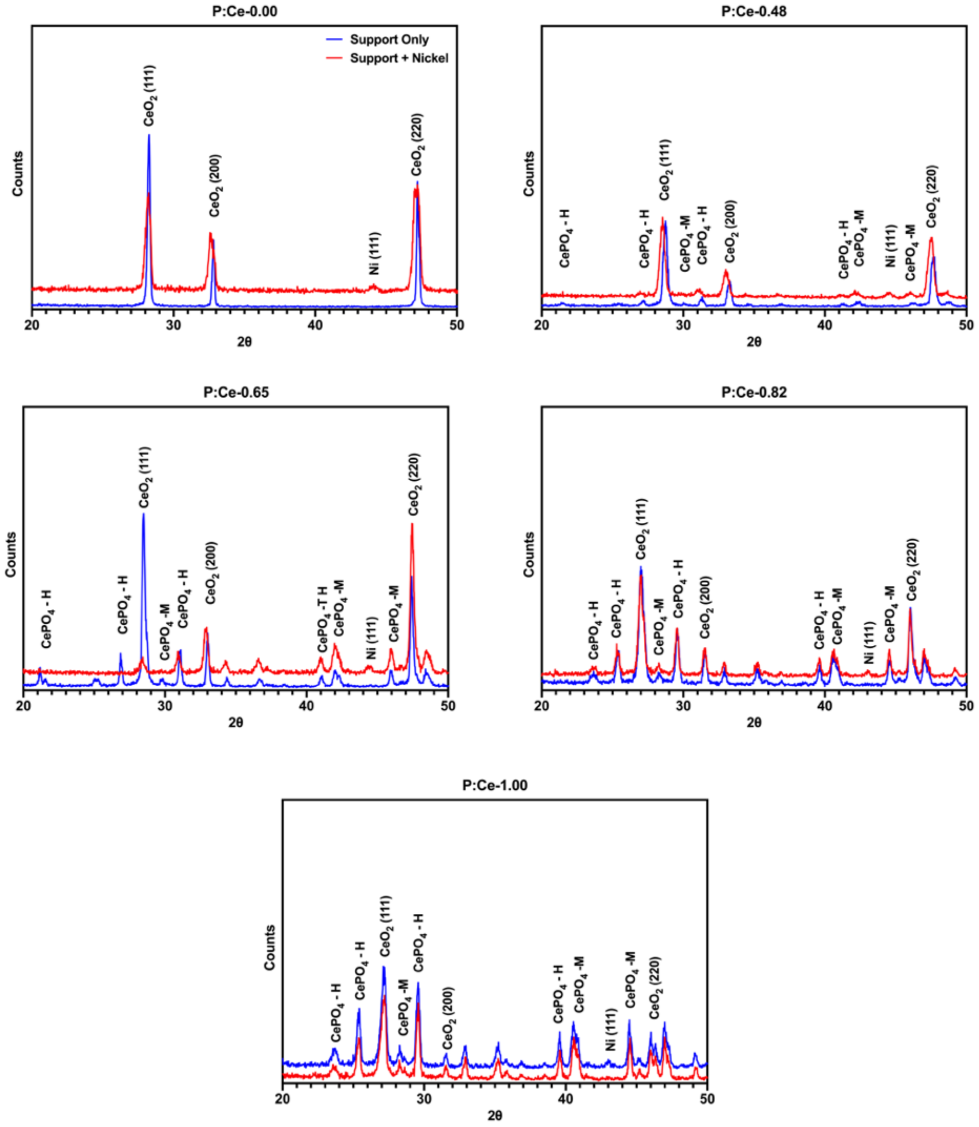

Figure S5. PXRD spectra for the Ni-CeO_2-$CePO_4$ photocatalyst of different P:Ce ratios.

Thermogravimetric Analysis

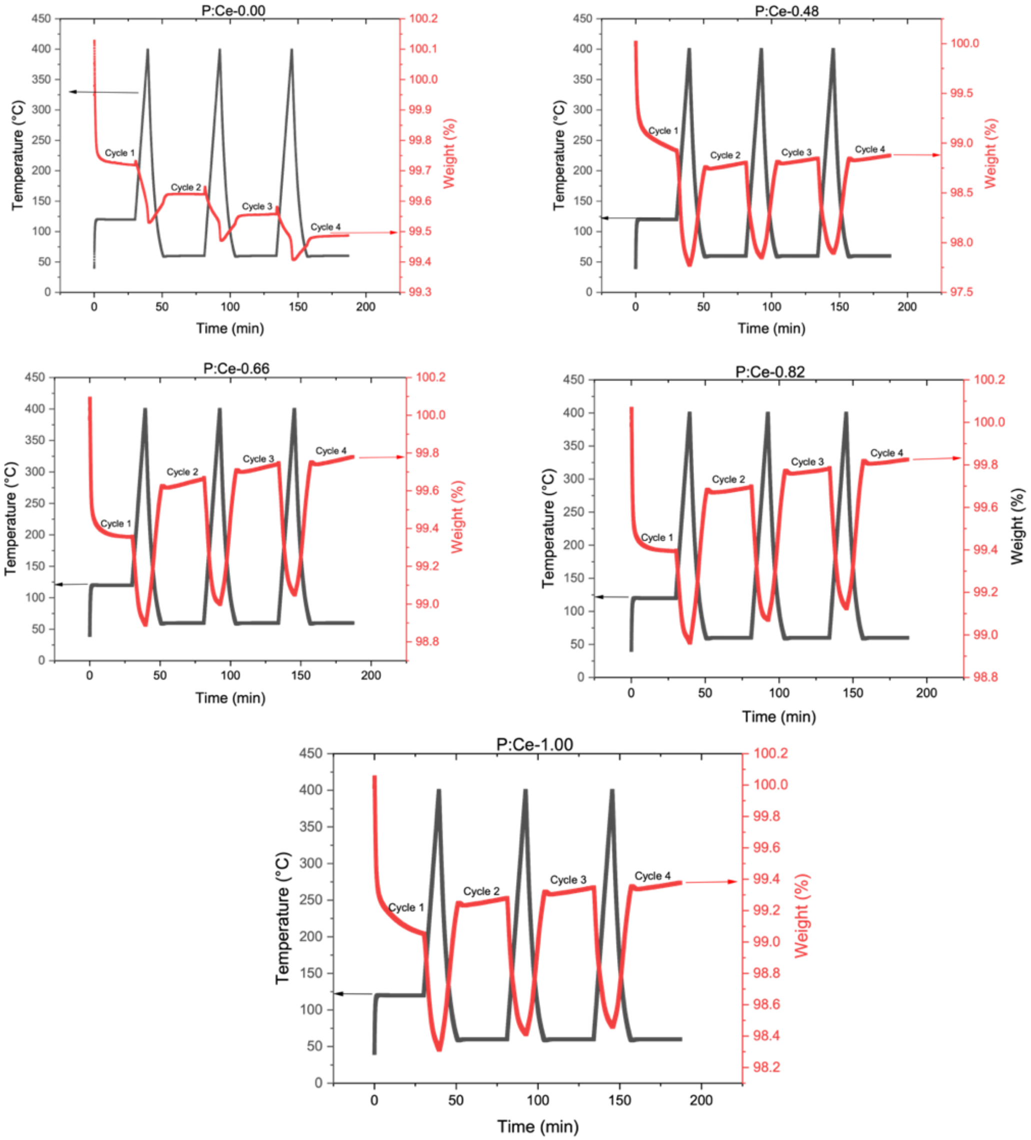

Figure S6. TGA for the Ni-CeO_2-$CePO_4$ photocatalyst of different P:Ce ratios.

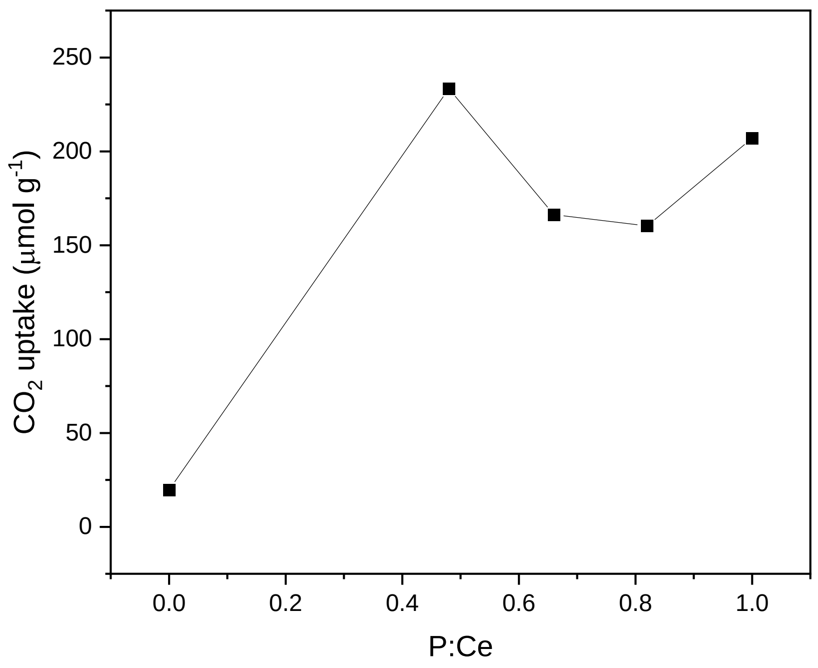

Figure S7. CO_2 uptake by TGA at 60 ºC with varying P:Ce ratio calculated from Figure S7

LED Lamp Spectra and Power

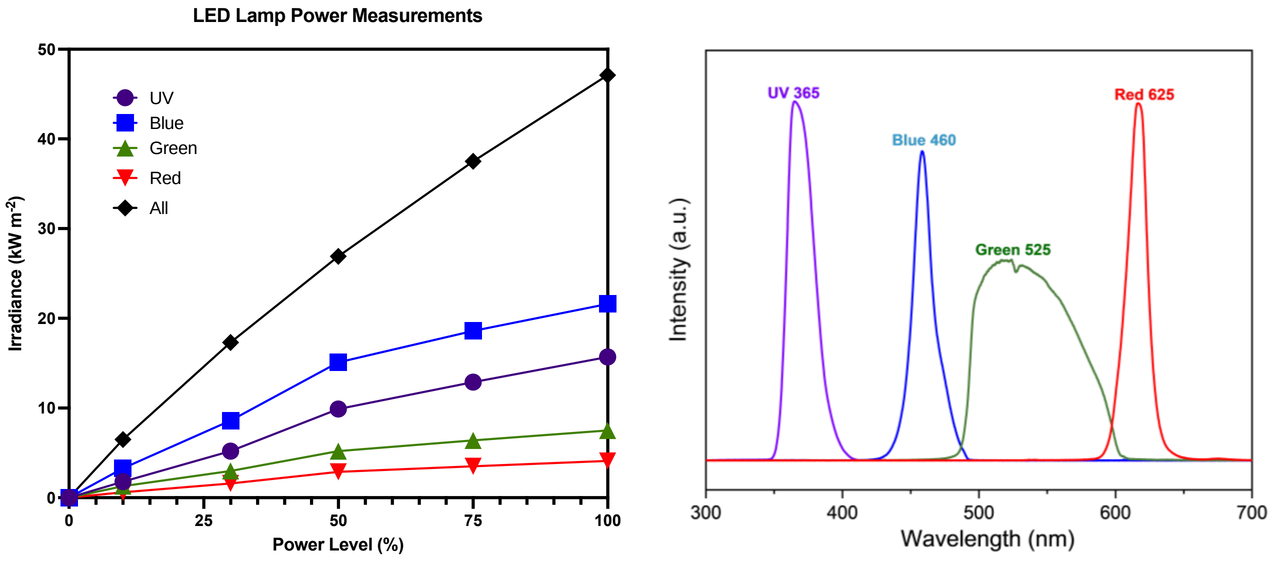

Figure S8. LED lamp power measurements along with LED lamp spectra.

DRIFTS Spectra

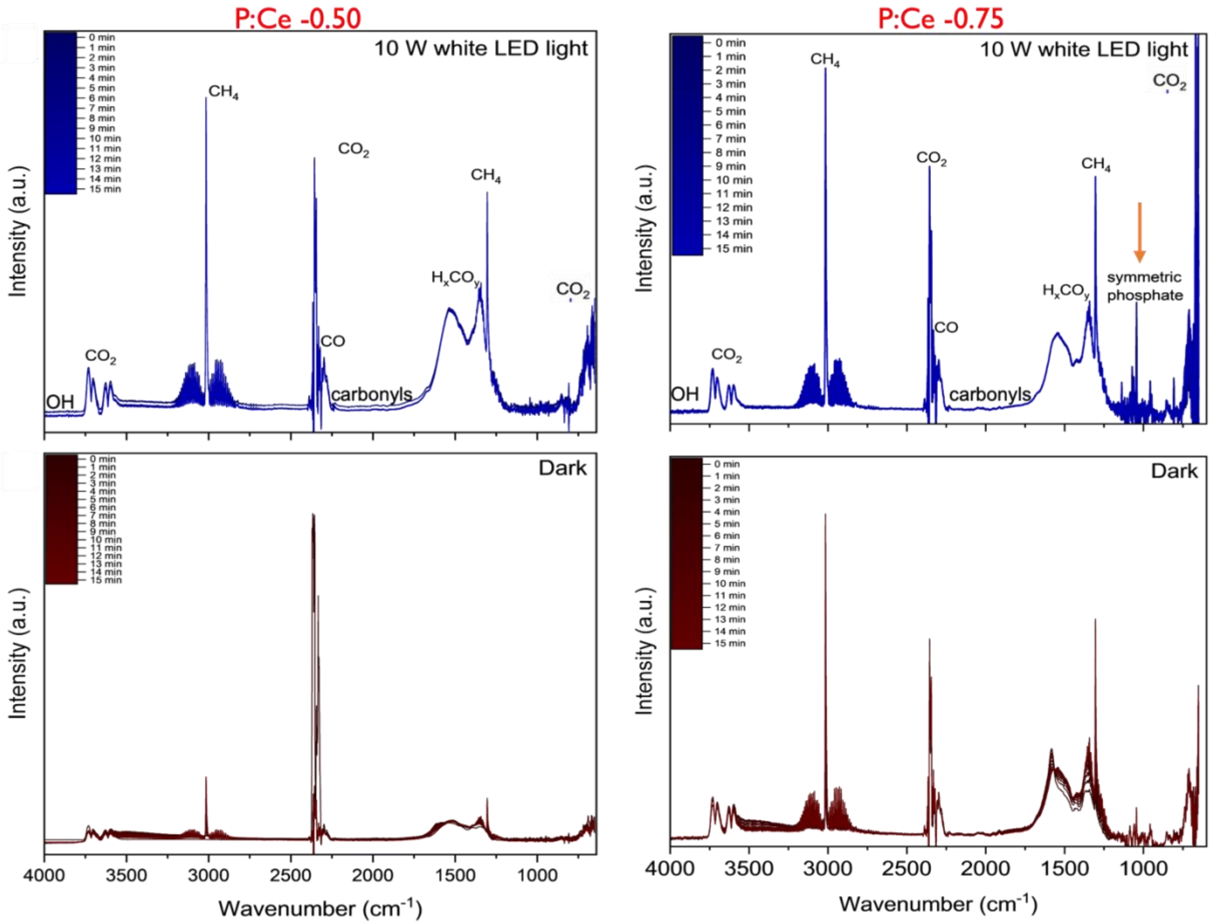

Figure S9. In-situ DRIFTS spectra measured during DRM reaction over the Ni-CeO_2-$CePO_4$ photo catalyst at the optimum conditions with varying P:Ce ratio

References

[1] Tavasoli, A., Preston, M., Ozin, G. Photocatalytic dry reforming: What is it good for? *Energy & Environ. Sci.* 14, 3098-3109 (2021)
[2] Han, B., Wei, W., Chang, L., Cheng, P., and Hu, Y.H. Efficient visible light photocatalytic CO_2 reforming of CH_4. 6, p. 494-497 (2015)
[3] Zhou, L., Martirez, J.M.P., Finzel, J., Zhang, C., Swearer, D.F., Tian, S., Robatjazi, H., Lou, M., Dong, L., Henderson, L. Christopher, P., Carter, E.A., Norlander, P. and Halas, N.J. Light-driven methane dry reforming with single atomic site antenna-reactor plasmonic photocatalysts. *Nature Energy* 5, 61-70 (2020)
[4] Shoji, S. Peng, X. Yamaguchi, A. Watanabe, R., Fukuhara, C., Cho, Y. Yamamoto, T. and Matsumura, S. and Yu, M. -. and Ishii, S. and Fujita, T. and Abe, H. and Miyauchi, M. Photocatalytic uphill conversion of natural gas beyond the limitation of thermal reaction systems. *Nature Catalysis*. 3, 148-153 (2020)
[5] Pan, F. Xiang, X., Deng,W., Zhao, H., Feng, X., Li, Y. A Novel Photo-thermochemical Approach for Enhanced Carbon Dioxide Reforming of Methane. *ChemCatChem*. 5, 940-945 (2018)
[6] Mao, M. Zhang, Q. Yang, Y. Li, Y. Huang, H. Jiang, Z. Hu, Q. and Zhao, X. Solar-light-driven CO_2 reduction by methane on Pt nanocrystals partially embedded in mesoporous CeO_2 nanorods with high light-to-fuel efficiency. *Green Chemistry*, 20, 2857-2869 (2018)
[7] Zhang, W., Mao, M., Li, Y., Yang, Y., Huang, H. Jiang, Z., Hu, Q., Wu, S., Xiujian, Z. Novel photoactivation promoted light-driven CO_2 reduction by CH_4 on Ni/CeO_2 nanocomposite with high light-to-fuel efficiency and enhanced stability. *Applied Catalysis B: Environmental.* 239, 555-564 (2018)
[8] Lorber, K., Zavašnik, J., Sancho-Parramon, J., Bubaš, M., Mazaj, M., Djinović, P., On the mechanism of visible light accelerated methane dry reforming reaction over Ni/CeO_{2-x} catalysts. *Applied Catalysis B: Environmental*. 301, 120745 (2022)
